# Water pipe smoking among public versus private university students in Ankara, Turkey: an online survey

**DOI:** 10.1186/s12889-022-13616-9

**Published:** 2022-06-25

**Authors:** Asena Caner, Hilal Özcebe

**Affiliations:** 1 Department of Economics, TOBB University of Economics and Technology, 06560 Ankara, Turkey; 2 Department of Public Health, Faculty of Medicine, Hacettepe University, 06230 Ankara, Turkey

**Keywords:** Water pipe smoking, Narghile, Shisha, Hookah, University student, Prevalence, Young adult, Turkey

## Abstract

**Background:**

Water pipe smoking has become a global public health problem as its popularity increased over time, especially among youth. The objective of our study was to estimate water pipe tobacco smoking prevalence and to assess socioeconomic factors associated with ever water pipe smoking by public and private university students in Ankara, Turkey.

**Methods:**

This descriptive study was based on a survey conducted among public (*n*=2685) and private (*n*=2485) university students via an online questionnaire on demographics and water pipe consumption patterns. For every student in the sample, a socioeconomic status index was calculated using principal component analysis. Binary logistic regressions for the outcome variable of ever-using water pipe yielded estimates of adjusted odds ratios (aORs) for the associated factors such as the respondent’s age, gender, university type, and socioeconomic status.

**Results:**

The prevalence of ever use of water pipe was 69.1% (95% CI: 67.2-70.9%) among private and 59.1% (95% CI: 57.2-60.9%) among public university students. A substantial share of ever users were current users (25.5% in private versus 21.6% in public, *p*=0.008). On average, private university students had higher socioeconomic status than public university students (for example, access to a car (51.7% versus 35.8%, *p*=0.008), financial support from family (71.5% versus 65.1%, *p*<0.001)), also demonstrated by a higher socioeconomic status index. Being a private university student (aOR 1.57, 95% CI: 1.38-1.79), older (aORs 1.50 to 2.39, *p*<0.001), male (aOR 2.36, 95% CI:2.06-2.70), as well as having greater financial resources, such as having access to a car (aOR 1.24, 95% CI:1.07-1.42), or having income support from family (aOR 1.32, 95% CI:1.13-1.54), were associated with ever-using water pipe. A higher SES index was significantly associated with higher odds of ever using water pipe among both private (aOR 1.13, 95% CI:1.06,1.20) and public university (aOR 1.12, 95% CI:1.06,1.19) students.

**Conclusions:**

Water pipe smoking was common in both public and private universities; however, private university students had higher odds of ever using water pipe. There is an urgent need to implement evidence-based interventions, taking into account the socioeconomic status of young adults, to prevent them from water pipe smoking.

**Supplementary Information:**

The online version contains supplementary material available at 10.1186/s12889-022-13616-9.

## Background

The consumption of tobacco in a water pipe (WP) (also known as narghile, hookah, or shisha) is a serious public health problem that is known to be associated with several adverse health outcomes such as respiratory diseases, bronchitis, oral cancer, lung cancer, low birth weight, metabolic syndrome, and cardiovascular diseases [1]. WP use originated in the Middle Eastern countries and has existed for centuries [2]. Unlike other tobacco products, WP is often used communally and in a prolonged time period [3]. As most of the consumption is intermittent, users rarely consider themselves under risk of addiction or facing severe health consequences [3].

WP smoking in young people is worrying because of the economic burden it can generate in the long term by reducing productivity and imposing health costs. Therefore, it is important to understand the correlates of WP use among youth. Unfortunately, WP smoking has increased its popularity among adolescents and youth in the world. According to Global Youth Tobacco Survey, in 34 of the 100 sites surveyed, the use of tobacco products other than cigarettes increased, which was largely attributed to rising WP use [4]. The prevalence of WP smoking is much higher in Eastern Mediterranean and European countries than in the other parts of the world, and also much higher among young people than adults. Studies conducted in Eastern Mediterranean countries reported the prevalence rate between 14.9% and 65.3% in years 2002 to 2014 [5].

In Turkey, tobacco control is an important part of public health policy. The first law in 1996 aimed to protect people from tobacco smoke in governmental buildings, and health and educational institutions. The ban was broadened in 2008 to include school premises, all tourism and hospitality workplaces, and commercial taxis. Then, the hospitality sectors started to promote WP service especially to young people and tobacco smokers at cafés or “WP cafés”. The industry used the common belief in the community that WP smoking is less harmful than cigarette smoking because the harmful particles of WP smoke is filtered into the water. They also offered WP with flavored tobacco to enrich taste and smell. Young people began to enjoy smoking WP with their friends for hours and WP cafés became popular locations for socialization [6–8]. The promotion of WP to young people has caused an increase in the prevalence of its use among young people. According to the Global Youth Tobacco Survey (2017) in Turkey, the percentage of WP ever-users was 24.6% among 13-15 year old adolescents (31.6% for boys and 17.5% for girls) [9]. Other studies in Turkey reported the prevalence of WP ever-use among university students as between 18.9% and 48% [10, 11].

In Turkey, there were 129 public (state) universities and 72 private foundation universities in the 2018-19 academic year [12]. Tuition fees of private universities are much higher than the fees of public universities. In the 2018-19 academic year, registration fees of public universities were between 60-125 USD, whereas the tuition of private foundation universities were between 7,873 - 11,135 USD [13] (quite high compared to per capita gross domestic product of Turkey, which was 9,792 USD in 2018 [14]). Therefore, students choose their higher education institutions depending on their socioeconomic background and their access to financial resources. It is already known that the prevalence of ever using WP has been rising among university students [15]. Starting university education brings new responsibilities in an unknown social environment and the type of the university attended is one of the main determinants of the social and economic environment of a student. Smoking behavior is affected by individual, social, institutional, and political factors. Being male and having a relative or friend who is a smoker are important determinants among adolescents and youth. In universities, the social environment as well as the financial resources of young people shape students’ preferences, lifestyle, and smoking habits [16, 17].

The objective of our study was to estimate WP tobacco smoking prevalence, study the patterns of WP smoking (initiation, frequency, reasons, and location of smoking), and to assess socioeconomic factors associated with ever WP smoking by public and private university students in Ankara, Turkey. The main contribution is to compare students in the two types of universities, namely private foundation universities and public universities.

## Methods

### Study design, setting, and participants

A cross-sectional (descriptive) online survey was conducted among university students in Ankara, the capital city of Turkey in the 2018-2019 academic year. In that year, there were 12 private and 7 public universities in Ankara. The number of university students in Ankara, the target population, was 26,781 male, 26,674 female in private universities, and 72,627 male, 80,607 female in public universities [12].

### Survey instrument

To collect data, a questionnaire (prepared in SurveyMonkey) was used. The questionnaire had 46 questions on sociodemographic characteristics, tobacco smoking habits, and knowledge-attitudes on smoking. (Please see the Supplementary Information for the questionnaire.) It was developed by the researchers by adopting the questions in internationally validated questionnaires (specifically, the Global Adult Tobacco Survey of the World Health Organization and CDC [18]). To further validate the questionnaire, the questions were reviewed by researchers experienced in tobacco control in Turkey, and also a pilot testing of the online survey was done to ensure that the questions were clear and the survey ran smoothly. It took on average 6-7 minutes to complete the questionnaire. Permission was obtained by the authors from the Ethics Committees of the respective universities prior to data collection.

### Sampling

It was aimed to obtain a sample that had representation of students enrolled in public and private universities in Ankara. Two private universities and one public university were invited to participate. One of the private universities was invited since it was the oldest one in Ankara and had large enrollment. The other two universities were invited because the researchers were associated with them. At the time of the survey, there were 11,014 (5,881 male and 5,133 female) and 5,245 (2,702 male and 2,543 female) undergraduate students in the two private foundation universities, and 35,331 (14,894 male and 20,437 female) undergraduate students in the public university.

The student affairs or related administrative departments of the universities were requested to send the invitation email to all enrolled students through registered student email addresses. The participant inclusion criterion was being an undergraduate student in one of the three universities. The invitation email included general information about the study and the link to the online questionnaire. Reminder emails were sent every 3-4 weeks. The survey remained open for almost 3 months. The students answered the questionnaire of the study on a voluntary basis; they were not provided with any incentives. Informed consent was obtained from all participating students; no student was under 18. The convenience (non-probability) sample, consisting of students who participated in the survey, included 1,362 and 1,215 students in the private universities, and 2,731 students in the public university. Out of a total of 51,590 students who were invited to the study 5,308 responded, which yielded a response rate of 10.3%.

### Measurement

#### Demographics and WP use pattern

Demographic characteristics data included gender and age. WP use status was determined based on ever using it, relying on the question “Have you ever smoked WP?”, since ever using WP is a major risk factor of using tobacco products in the future. If participants had ever smoked WP, their patterns of use (i.e., age at initiation, location of use, sharing WP, WP cafes close to university, using in the last month, and reasons for using) and the amount of spending on WP were surveyed.

#### Indicators of socioeconomic status

Family income or wealth were not asked in the survey, because of the difficulty of precisely measuring these variables in online surveys with voluntary participation. Instead, the survey included three other questions to help assess socioeconomic status (SES): Whether the student had access to a car (regardless of ownership); Living arrangement of the student (four categories: living in a dormitory, living alone (outside of dormitory), living at home with family, or sharing the residence with friends); and Source of income of the student (three categories: Family, scholarship, work). In the analyses, the binary (dummy) variable “Has a car” and the categorical variables “Living arrangement” and “Source of Income” were used as indicators of SES of students.

In addition, to summarize the information in the three variables described above, a SES index was generated. The SES index [19] was developed by calculating the first principal component of eight binary variables: Has a car, Lives with family, Lives in dormitory, Lives alone, Has roommate(s), Income source: Family support, Income source: Scholarship, and Income source: Work. Using the factor scores from the first principal component as weights, a SES index was constructed for each student in the dataset.

### Statistical methods

Stata/MP 15.1 was used to perform statistical analyses. Descriptive statistics were reported for never and ever smoking WP by students in private and public universities. Among ever users of water pipe, descriptive characteristics on the patterns of water pipe use were presented. To test whether students in private universities had the same prevalence rate or similar characteristics as those in public universities, p-values from chi-square tests were used. Binary logistic regressions, where the outcome variable was ever using WP, were used to estimate adjusted odds ratios (aOR) with 95% confidence intervals (CIs) for the associated factors (gender, age, having access to a car, living arrangement, source of income). Binary logistic regression was estimated also for the associated factors of gender, age, and SES index. To compare aORs between public and private university students, tests of equality of the aORs were conducted. Regressions were estimated in samples of private and public university students separately, as well as in the pooled sample, where a binary (dummy) variable for being a private university student was added as another associated factor.

## Results

A total of 2,485 private and 2,685 public university students answered the question on ever smoking WP. Students from different schools and departments of the universities participated in the survey. About 36% were from the School of Engineering, 27% from the School of Economic and Administrative Sciences, 15% from the School of Science and Letters, 6% from the School of Fine Arts, Design, and Architecture, 4% from the Law School, 3% were from the School of Nursery, 2.9% were from the School of Dentistry, and 2.6% were from the School of Pharmacy.

As shown in Table 1, the ever-smoking prevalence of WP was 69.1% (95% CI: 67.2-70.9) in private universities and 59.1% (95% CI: 57.2-60.9) in public university. A breakdown of the sample by demographic characteristics revealed higher prevalence of ever use of WP among private university students compared to public university students (males, 76.3% versus 72.7%, *p*=0.045; females 61.4% versus 49%, *p*<0.001; in age groups the corresponding p-values were <0.001 in ages 18-19, 20-21, and 22-23). In most of the socioeconomic groups, the ever-smoking prevalence of WP was higher in private universities than in public university (for example, among students with access to a car (*p*=0.008), among those who live alone (*p*=0.017), among those who receive family support (*p*=0.001).

**Table 1:** Demographics of survey participants by university type and WP use status

	Private				Public				
	Ever	Never	All		Ever	Never	All		
	%	%	n	%	%	%	n	%	***p***-value
**Gender**									
Male	76.3	23.7	1,289	51.9	72.7	27.3	1,140	42.5	**0.045**
Female	61.4	38.6	1,196	48.1	49.0	51.0	1,545	57.5	**<0.001**
**Age group**									
18-19	59.2	40.8	485	19.9	45.4	54.6	377	14.6	**<0.001**
20-21	66.4	33.6	1,010	41.4	56.7	43.3	1,057	40.8	**<0.001**
22-23	76.3	23.7	710	29.1	61.5	38.5	799	30.8	**<0.001**
24 or older	80.9	19.1	236	9.7	74.3	25.7	358	13.8	0.060
**Access to a car**									
Yes	73.1	26.9	1,126	51.7	67.7	32.3	895	35.8	**0.008**
No	64.3	35.7	1,052	48.3	54.4	45.6	1,607	64.2	**<0.001**
**Living arrangement**									
In dormitory	65.5	34.5	837	36.3	49.7	50.3	927	36.5	**<0.001**
Lives alone	90.1	9.9	131	5.7	79.6	20.4	137	5.4	**0.017**
Lives with family	68.0	32.0	1,189	51.6	60.7	39.3	1,178	46.4	**<0.001**
Has roommate(s)	82.2	17.8	146	6.3	72.9	27.1	295	11.6	**0.031**
Source of income*									
Family support	70.6	29.4	1,716	71.5	58.2	41.8	1,706	65.1	**<0.001**
Scholarship	60.4	39.6	490	20.4	51.6	48.4	543	20.7	**0.004**
Work	78.3	21.7	92	3.8	75.0	25.0	252	9.6	0.532
**Total**	69.1	30.9	2,485	100.0	59.1	40.9	2,685	100.0	**<0.001**
**95% CI on ever WP use prevalence**	[67.2-70.9]				[57.2-60.9]				

*Notes*: The *p*-value refers to the chi-square test where the null hypothesis is no relationship between the type of the university and WP use status. (*) More than one income source could be selected

Table 2 depicts that WP was most often used outside of home (at a narghile café or at other cafés, restaurants, or tea houses). More than 85% of the students usually shared their WP among the students in both private and public universities. Compared to public university students, more opportunities (a higher number of WP offering venues) existed for private university students close to their university (*p*<0.001). The prevalence of WP use within the last month was higher among private university students (14.8% versus 8.7%; *p*<0.001). Among ever-users of WP, the prevalence of current WP use was higher among private university students (25.5% versus 21.6%; *p*=0.008).

**Table 2:** Patterns of WP smoking among respondents who have ever smoked WP

	Private University		Public University		***p***-value
	n	%	n	%	
**Age at first use**					0.133
Younger than 14	171	10.1	133	8.4	
Ages 14-18	1,074	63.2	990	62.6	
Older than 18	454	26.7	459	29.0	
**Location of WP use**					0.303
At home only	21	3.8	26	5.7	
Outside of home only	489	87.9	395	87.0	
Both at home and outside of home	46	8.3	33	7.3	
**WP sharing (usually)**					0.101
No	78	14.4	48	10.9	
Yes	465	85.6	394	89.1	
**Number of WP venues close to university**					**<0.001**
None	224	13.5	359	23.2	
1-3 venues	677	40.8	593	38.3	
4 or more venues	758	45.7	598	38.6	
**Use within the last month**					**<0.001**
Yes	253	14.8	137	8.7	
No	1,454	85.2	1,445	91.3	
Reasons for WP use*					
Enjoy the aroma	1293	75.3	1197	75.5	0.911
Pleasurable	889	51.8	677	42.7	**<0.001**
Facilitates socialization	767	44.7	524	33.0	**<0.001**
Smoke does not hurt throat	539	31.4	492	31.0	0.818
Can be shared with friends	533	31.0	397	25.0	**<0.001**
Makes conversation more fun	478	27.8	360	22.7	**0.001**
Part of traditional culture	339	19.7	249	15.7	**0.002**
Makes nice visual in social media	306	17.8	285	18.0	0.912
Nice ambience and food	304	17.7	220	13.9	**0.003**
Shares in social media invoke curiosity	185	10.8	143	9.0	0.091
**Current users of WP (among ever users of WP)**	438	25.5	342	21.6	**0.008**

*Notes*: Sum of n’s may differ across categories since not all questions were answered by all participants

(*) More than one reason could be selected

The *p*-value refers to the chi-square test where the null hypothesis is no relationship between the type of the university and the sets of variables reported in the rows of the table

Students enjoyed WP for several reasons. The sensory charms of WP were important for youth. Compared to public university students, private university students found WP more enjoyable in many respects: Being pleasurable (51.8% versus 42.7%, *p*<0.001), facilitating socialization (44.7% versus 33.0%, *p*<0.001), can be shared with friends (31.0% versus 25.0%, *p*<0.001), makes conversation more fun (27.8% versus 22.7%, *p*=0.001), part of traditional culture (19.7% versus 15.7%, *p*=0.002), nice ambience and food in the venue (17.7% versus 13.9%, *p*=0.003) Table 2.

The binary logistic regression estimates were obtained for students who answered all questions that were of interest to this study (2184 private university students and 2352 public university students). Table 3, which presents the estimates obtained separately for private and public university students, showed that in both types of universities, being male (aORs 2.23 and 2.50) and being older (aORs between 1.33 and 2.48) were positively associated with ever use of WP. In the public university, having access to a car was associated with higher odds (aOR 1.37) of ever using WP. In both types of universities, compared to those living in the dormitory, students who lived alone (aORs 3.19 and 2.13) or had roommate(s) (aORs 1.92 and 1.67) had higher odds of ever using WP. Living with family was associated with higher odds in the public university (aOR 1.26). Compared to living on a scholarship, being financially supported by the family was associated with higher odds of ever WP use in the private universities (aOR 1.66). A test of the equality of the aORs in private and public university regressions showed that private university students who relied financially on family support were more likely to ever use WP, relative to public university students (at 5% significance level) (results not shown in the table). The last column in Table 3 shows the estimates for the entire sample of students. Being in a private university was associated with higher odds of ever using WP (aOR 1.57), after controlling for the other associated factors.

**Table 3:** Ever smoked WP: Logistic regression estimates (aOR [95% CI])

	Private University Students	Public University Students	All Students
**Private university**			1.57***
			[1.38,1.79]
**Gender**			
Female	1	1	1
Male	2.23***	2.50***	2.36***
	[1.83,2.71]	[2.08,3.01]	[2.06,2.70]
**Age group**			
Ages 18-19	1	1	1
Ages 20-21	1.33**	1.67***	1.50***
	[1.04,1.70]	[1.28,2.16]	[1.26,1.79]
Ages 22-23	2.31***	1.87***	2.04***
	[1.75,3.04]	[1.42,2.47]	[1.68,2.47]
Ages 24 or older	2.41***	2.48***	2.39***
	[1.57,3.69]	[1.74,3.53]	[1.83,3.12]
**Access to a car**			
No car	1	1	1
Has a car	1.12	1.37***	1.24***
	[0.91,1.37]	[1.12,1.67]	[1.07,1.42]
**Living arrangement**			
Lives in dormitory	1	1	1
Home with family	1.00	1.26**	1.15*
	[0.80,1.24]	[1.03,1.53]	[1.00,1.33]
Lives alone	3.19***	2.13***	2.56***
	[1.69,6.01]	[1.31,3.47]	[1.75,3.74]
Has roommate(s)	1.92***	1.67***	1.71***
	[1.19,3.10]	[1.22,2.29]	[1.32,2.21]
**Source of income**			
Scholarship	1	1	1
Income source: Family Support	1.66***	1.13	1.32***
	[1.30,2.11]	[0.91,1.39]	[1.13,1.54]
Income source: Work	1.75*	2.10***	2.07***
	[0.94,3.25]	[1.37,3.22]	[1.46,2.94]
**Observations**	2184	2352	4536

*Notes*: The first two columns show estimates of adjusted odds ratios (adjusted for all associated factors variables listed in the table) separately for private and public university students, in multivariable binary logistic regressions. The last column shows the estimates for the entire sample, adding the “Private university” dummy (binary) variable to the regression. 95% CI shown in square brackets. Reference categories: Female, Ages 18-19, No car, Lives in dormitory, and Scholarship. *** *p*<0.01, ** *p*<0.05, * *p*<0.10

In the calculation of the SES index, the first principal component (with the largest eigenvalue of 2.12) was used. It had positive factor scores on four variables (has a car (0.3281), lives with family (0.4979), lives alone (0.0231), and receives family support (0.4370)); therefore, these variables were thought to be associated with higher SES. The other four variables had negative factor scores: lives in dormitory (-0.4904), has roommate(s) (-0.0067), work as source of income (-0.0051), scholarship as source of income (-0.4609); therefore, they were thought to be associated with lower SES.

It was found that private university students, on average, had higher SES than public university students. On average, the SES index was statistically significantly higher for private university students (0.0966, standard error 0.0302) than for public university students (-0.0893, standard error 0.0265). A t-test for equality of means yielded a t-statistic of 4.62, leading to the rejection of equal means in private and public universities. The SES index took higher values for those who had a car, lived with family, lived alone, or received financial support from family. Consistent with this result, Table 1 shows that, on average, a higher share of private university students had access to a car (51.7% versus 35.8%) and relied on their families for financial support (71.5% versus 65.1%), compared to public university students. Moreover, among current users of WP who revealed the amount of their spending on WP (*n*=752), average monthly spending on WP was statistically significantly higher among private university students (42 TL) than among public university students (29.6 TL) (not tabulated).

Table 4 presents the estimates obtained from a binary logistic regression that replaced the variables that were used to assess SES with the SES index. A higher SES index was significantly associated with higher odds of ever using WP in both private university (aOR 1.13, 95% CI [1.06,1.20]) and public university (aOR 1.12, 95% CI [1.06,1.19]). As in Table 3, male and older students were more likely to ever use WP.

**Table 4:** Ever smoked WP: Logistic regression estimates (aORs [95% CI]), SES index used as an associated factor

	Private University Students	Public University Students	All Students
**Private university**			1.54***
			[1.35,1.75]
**Gender**			
Female	1	1	1
Male	2.31***	2.77***	2.53***
	[1.91,2.80]	[2.31,3.32]	[2.22,2.88]
**Age group**			
Ages 18-19	1	1	1
Ages 20-21	1.40***	1.78***	1.58***
	[1.10,1.79]	[1.37,2.30]	[1.32,1.88]
Ages 22-23	2.45***	2.13***	2.24***
	[1.87,3.22]	[1.62,2.79]	[1.85,2.70]
Ages 24 or older	2.81***	3.29***	3.07***
	[1.88,4.22]	[2.34,4.62]	[2.37,3.97]
**SES index**	1.13***	1.12***	1.13***
	[1.06,1.20]	[1.06,1.19]	[1.08,1.18]
**Observations**	2184	2352	4536

*Notes*: The SES index was calculated using the first principal component of eight binary variables (taking values of zero or one): Has a car, Lives with family, Lives in dormitory, Lives alone, Has roommate(s), Income source: Family support, Income source: Scholarship, Income source: Work. The first principal component had positive loadings on four variables (has a car, lives with family, lives alone, and receives family support). These four variables can be thought to be associated with higher socioeconomic status. The other four variables had negative factor loadings. The mean (and standard error) values of the index for private and public university students were 0.0966 (0.0302) and -0.0893 (0.0265), respectively. A two-sample t-test for difference in means yielded a t-statistic of -4.62; therefore, the null hypothesis of equality of means was rejected

The first two columns in the table show estimates of adjusted odds ratios (adjusted for all associated factors variables listed in the table) from binary logistic regressions, separately for private and public university students. The last column shows the estimates for the entire sample, adding the “Private university” dummy (binary) variable to the regression. 95% CI are shown in square brackets. ***p<0.01

## Discussion

This study offers the first evidence that the prevalence of ever using WP was higher among private than public university students. In public universities 59.1% of students were ever users of WP; whereas among private university students 69.1% were ever users of WP. A substantial share of ever users of WP were current users of WP at the time of the survey, with a higher share reported among private than public university students.

In this study, it was found that SES was an important factor associated with ever using WP. Moreover, SES was significantly higher for private university students than for public university students. Regression analyses showed that higher SES (as measured by the SES index) was statistically significantly associated with higher odds of ever using WP. It is also important that, even after controlling for the SES index, private university students still had higher odds of ever using WP.

Several studies have reported that the prevalence of WP use among youth is quite high in the Middle Eastern countries and has been rising in the western part of the world as well [3, 5, 16, 20–22]. As explained in the Introduction, the related studies in the literature reported the prevalence of WP ever-use among university students as between 18.9% and 48% in Turkey [10, 11]. Our results indicate much higher prevalence rates than declared in these international and earlier national studies, and they show that WP smoking has become more popular in the country. This study confirmed the earlier studies that the sensory charms of WP still strongly contributed to its popularity among university students [20, 21]. Moreover, our findings emphasized once again that WP facilitated socialization among university students, it is shared with friends, and makes conversation more fun [22, 23].

Evidently, WP retail venues (cafés, tea houses) were located in spots popular among youth and were in close proximity to students (around campuses), providing easy access. We found that such places were more abundant in locations close to private than public university students, because WP smoking at these venues can be quite costly. As explained before, private universities charge a substantial amount of tuition; therefore, it is not surprising that private university students come from more affluent families [24]. It is easier for students with more financial resources to afford WP. Two more findings suggested that WP use was associated with higher financial resources: First, a higher proportion of users in public than private universities usually shared WP (as opposed to consuming it alone); and, secondly, the average monthly spending on WP was statistically significantly higher among private than public university students.

This study found that the prevalence of ever using WP was higher among men than women, as in the earlier national studies on adolescents and youth [10, 11]. The relative popularity among men can be related to the perception of smoking, in general, and WP smoking, in particular, as a traditional masculine behavior. Among women university students, although the rates estimated in Turkey were lower than those in Eastern Mediterranean countries [25], they were still quite high (for example, 20% were current users and 35% were ever users [10]). On the other hand, global statistics indicate that the popularity of WP smoking has risen faster among women than men [26–28]. In some countries, WP smoking has become the leading form of tobacco use among young women [29, 30]. WP smoking among women is perceived as a sexy and charming behavior [31, 32] and may also be viewed by females as a sign of social status, since it is viewed as luxurious and available only to those who can afford it [23]. As in other countries, availability and affordability of WP cafés in Turkey may contribute to WP smoking, especially among young women [28, 33, 34], who may feel emancipated and empowered by the capability of participating in a traditionally male-dominant environment [33, 35, 36].

The findings of this research should be interpreted in light of several limitations: The sample included only university students; therefore, non-student young adults were not covered. The sample was a convenience sample with participants recruited from three universities in Ankara. Although the study benefitted from a large sample, it might not be representative of university students in the country. Moreover, since participation was voluntary and the topic of the survey might have been more interesting to ever-smokers of WP, a larger share of ever-smoker than never-smoker students might have responded to the survey, leading to an overestimation of the prevalence rates. Another point is that Ankara is the capital city with a higher than average per capita income and greater availability of outlets where the youth can access WP. In smaller cities, the consumption pattern might be different. Also, the survey did not cover tobacco consumption in the family or the city where the student attended high school, both of which might play a role in initiation.

## Conclusions

Both private university and public university students had substantial rates of WP smoking prevalence. WP smoking was associated with higher financial resources and higher SES. The results highlight the need for stricter regulations to curb WP use among university students.

## Supplementary Information

**Additional file 1.**

## Data Availability

The datasets generated and analyzed, as well as the questionnaire, are available from the corresponding author upon request.

## Abbreviations

aOR: Adjusted odds ratio
CI: Confidence interval
p: p-value
