# Supplementary material for: Water pipe smoking among public versus private university students in Ankara, Turkey: an online survey
Source: BMC Public Health. 2022 Jun 25;22:1256. doi: 10.1186/s12889-022-13616-9 (PMC9233815; doi:10.1186/s12889-022-13616-9)
Supplement: Supplementary file 1 — Additional file 1. [file 12889_2022_13616_MOESM1_ESM.docx]

# Additional file 1:

# Survey Questionnaire

The questions in the survey were as follows:

Q0. Please read the informed consent form. If you agree to participate in this study, please proceed; otherwise, please quit.

Q1. What is your gender? (Female; male)

Q2. What is your birth year?

Q3. a. Which school are you enrolled in? (Engineering; Economics and Administrative Sciences; Management; Arts and Sciences; Fine Arts, Design, and Architecture; Law; Education; Medicine; Dentistry; Nursing; Sports Science)

b. When did you start studying at this school ?

Q4. What is your Grade Point Average (GPA) score? (0-0.50; 0.51-1.00; 1.01-1.50; 1.51-2.00; 2.01-2.50; 2.51-3.00; 3.01-3.50; 3.51-4.00)

Q5. Have you ever smoked cigarettes? (No, never; Yes, tried but did not continue; Yes, but quit; Yes, a current smoker)

Q6. How old were you when you smoked your first cigarette? (Younger than 14; 14-18; older than 18; never smoked)

Q7. How often do you smoke? (On a daily basis; not daily but occasionally; not a current smoker)

Q8. Had you been smoking on a daily basis or occasionally in the past? (Smoked daily; smoked occasionally; only tried but did not continue; never smoked)

Q9. How many cigarettes do you smoke per day? (10 or fewer; 11-20; 21-30; 31 or more; I do not smoke)

Q10. How much money do you spend on smoking per month? (0-10 TL; brackets of 10 TL until 300 TL; brackets of 50 TL from 300 to 500 TL; more than 500 TL; I do not smoke)

Q11. Do you think that you would consume more cigarettes if there was no smoking ban in closed areas? (Yes; No; I do not smoke)

Q12. When do you smoke your first cigarette of the day (or when did you smoke your first cigarette of the day in the past?)? (Within 5 min; in 6-30 min; in 31-60 min; in more than 60 min; I do not smoke)

Q13. Have you tried to quit smoking in the last twelve (12) months? (Yes; No; I do not smoke)

Q14. Have you ever smoked a water pipe? (Yes, a current user; Yes, tried but did not continue; No, never)

Q15. How old were you when you first used water pipe? (Younger than 14; 14-18; older than 18; never smoked water pipe)

Q16. Did you smoke water pipe in the last thirty (30) days? (Yes; No)

Q17. Do you smoke water pipe regularly? (Yes; No)

Q18. What often do you use water pipe? (Once in 2 months or less often; once a month; 2-3 times a month; once a week; twice or more often in a week; I do not smoke water pipe)

Q19. How much money do you spend per month on water pipe? (0-20 TL; 20-40; 40-60; 60-80; 80-100; 100-150; 150-200; 200-250; more than 250 TL; I do not smoke water pipe)

Q20. Have you tried to quit water pipe smoking in the last twelve (12) months? (Yes; No; I do not smoke water pipe)

Q21. Where do you usually use water pipe? Mark all that apply. (At home; at a water pipe café; at a café; at a restaurant; at a tea house; at a tea garden; I do not smoke water pipe)

Q22. Do you usually use the water pipe alone, or are you sharing it? (Alone; share with one person; share with 2 persons; share with 3 persons; share with 4 or more persons; I do not smoke water pipe)

Q23. In how many different places (venues) can you smoke water pipe close to your university? (None; one; 2-3; 4-5; more than 5)

Q24. Why do you think people use water pipes? (Enjoy the aroma; pleasurable; facilitates socialization; smoke does not hurt throat; can be shared with friends; makes conversation more fun; part of traditional culture; makes nice visual in social media; nice ambience and food served at the venue; shares in social media invoke curiosity)

Q25. How do you cover your living expenses? What is your primary source of income? (Family support; Scholarship/fellowship; Work; I do not want to say)

Q26. Where do you live when you are studying in college? (In dormitory; live alone; live with family; live with roommate(s))

Q27. Which of the following statements best describes the sufficiency of your income? (not sufficient (cannot pay for expenses); barely sufficient (can only pay for expenses but cannot save); sufficient (can pay for expenses and can save))

Q28. Do any of your friends smoke cigarettes? (All of them; most of them; some of them; none of them)

Q29. Does your girl/boyfriend (or your ex-girlfriend/boyfriend) smoke cigarettes? (Yes; no; not applicable)

Q30. Do any of your friends smoke water pipes? (All of them; most of them; some of them; none of them)

Q31. Does your girl/boyfriend (or your ex-girlfriend/boyfriend) smoke water pipe? (Yes; no; not applicable)

Q32. Do you agree with the statement "I would use more cigarettes if I had more money"? (Absolutely agree; agree; no opinion; do not agree; absolutely disagree)

Q33. Do you agree with the statement "I would smoke more if the cigarettes were cheaper"? (Absolutely agree; agree; no opinion; do not agree; absolutely disagree)

Q34. Do you agree with the phrase "I would use water pipe more often if I had more money"? (Absolutely agree; agree; no opinion; do not agree; absolutely disagree)

Q35. Do you agree with the phrase "I would use water pipe more often if the it was cheaper"? (Absolutely agree; agree; no opinion; do not agree; absolutely disagree)

Q36. Are there any legal regulations for indoor water pipe smoking? (yes, smoking water pipe in indoor areas is prohibited; no, smoking water pipe in indoor areas is not prohibited; do not know)

Q37. Which of the following is correct about the relative harm of cigarettes and water pipe smoking? (Equally harmful; water pipe is more harmful; cigarettes are more harmful; do not know)

Q38. Have you encountered a warning such as "Harmful to health" etc. in the places where water pipe is used? (Yes; no; did not pay attention; have never been at a place where water pipe is used)

Q39. Where are you exposed to secondhand cigarette smoke? Mark all that apply. (Café/restaurant; vehicles of transportation; open areas; business places/offices; at home; none)

Q40. Where are you exposed to secondhand water pipe smoke? Mark all that apply. (Café/restaurant; vehicles of transportation; open areas; business places/offices; at home; none)

Q41. Do any of your acquaintances have any of the following illnesses that are caused by tobacco products use? Mark all that apply. (Oral odor and reeling in teeth; heart and vascular diseases; chronic lung disease (bronchitis, COPD, etc.); gastritis, ulcer, and stomach cancer; skin wrinkles, skin cancer; blockage in veins and associated paralysis; prostate cancer; preterm birth in pregnancy and consequently various developmental disorders in child, and postpartum discontinuation; cervical cancer; bladder cancer; I do not know anyone who uses tobacco products; I know people who use tobacco products, but I do not know if they have a disease)

Q42. Do you have any chronic illnesses? (Yes; no; I do not know)

Q43. Have you ever heard of a product called electronic cigarette? (Yes; no)

Q44. Have you ever used electronic cigarettes? (Yes; no)

Q45. Do you know someone who uses electronic cigarettes? (Yes; no)

Q46. Do you have a car that you can use (even without having ownership)? (Yes; no)
